# Measuring energy expenditure in Göttingen Minipigs using indirect calorimetry: validation and methodological considerations

**DOI:** 10.1186/s42826-024-00233-3

**Published:** 2025-02-21

**Authors:** Simon K. Bredum, Julie Jacobsen, Susanna Cirera, Berit Ø. Christoffersen

**Affiliations:** 1 Integrated Physiology Research, Novo Nordisk A/S, Måløv, Denmark; 2 Department of Animal Welfare and Disease Control, University of Copenhagen, Frederiksberg, Denmark

**Keywords:** Energy expenditure, Indirect calorimetry, Göttingen Minipigs, Dinitrophenol, Glucagon receptor agonist, Melanocortin 4 receptor agonist

## Abstract

**Background:**

Obesity affects nearly a billion people globally and is associated with various health consequences. Current anti-obesity medications primarily target appetite, but drug candidates that modulate energy expenditure (EE) and substrate utilization based on respiratory exchange ratio (RER) are also essential to continuously improve the treatment modalities for people living with obesity. Selecting appropriate animal models and methods is crucial to improving translational value in preclinical research. While pig obesity models provide a relevant alternative to rodent models due to their similarities to humans, little is known about the assessment and translatability of EE in pigs. The aim of this study was to evaluate the translatability of minipigs for assessing the effect of EE-modulating drugs using indirect calorimetry and three positive control compounds that have known effects on EE and/or RER in humans. The study consisted of five sub-studies: Sub-study 1 assessed EE and RER based on sex (male/female) and diet (chow/high-fat diet) with and without correction for body composition; Sub-studies 2–4 evaluated changes in EE and RER after treatment with three positive control compounds: 2,4-dinitrophenol, DNP; a glucagon receptor agonist, GCG-RA; and a melanocortin receptor 4 agonist, MC4-RA; and sub-study 5 established three predictive equations for resting metabolic rate.

**Results:**

Sub-study 1 resulted in detectable differences in EE and RER based on diet/body sizes (*P*-value < 0.0001), while EE adjusted for body composition resulted in differences based on sex (*P*-value < 0.0001). Sub-studies 2–4 revealed that the three pharmacological interventions known to affect EE in humans, DNP, GCG-RA, and MC4-RA, showed similar effects in the Göttingen Minipigs by significantly increasing EE by 26.1% (*P*-value: 0.0014), 21.3% (*P*-value: 0.0491), and 25.4% (*P*-value: 0.0013), respectively, emphasizing the translational value of the model. In sub-study 5, three predictive equations were established for RMR based on body composition, demographic and anthropometric measurements, and the most accurate equation based on all variables. All three equations demonstrated acceptable accuracy (adjusted R^2^: 0.73–0.85).

**Conclusions:**

The present study qualifies the use of Göttingen Minipigs for investigating EE in preclinical research and provides a framework for conducting such research.

**Supplementary Information:**

The online version contains supplementary material available at 10.1186/s42826-024-00233-3.

## Background

Obesity is a chronic disease that can impair health, and in 2022, it was estimated to affect nearly one billion adults globally [1]. Lifestyle interventions such as dietary restriction and exercise are still important approaches to treating obesity. New incretin-based therapies that target appetite centers in the brain have induced a significant weight loss of more than 20% [2]. Pharmacological interventions targeting energy expenditure (EE) may adequately supplement the current treatments and induce an even greater and more sustainable weight loss. Several drugs aimed at increasing EE have entered preclinical and clinical development, but most have failed because they lack efficacy or have safety issues [3]. Drugs that affect EE in humans include the mitochondrial uncoupler 2,4-dinitrophenol (DNP) [4], native glucagon [5–7], and the melanocortin 4 receptor agonist (MC4-RA) RM-493 [8]. The high attrition rate highlights the importance of evaluating the efficacy and safety of EE-modulating drug candidates in relevant and translational preclinical animal models. Translating the research on EE from animal models to humans is challenging, however. The commonly used rodent models, unlike humans, have a substantial amount of brown adipose tissue (BAT) that can be highly metabolically active, affect EE, and influence the treatment effect of a drug on EE [9, 10]. The resemblances in anatomy, size, and metabolic rate between humans and pigs emphasize the potential for the greater translational relevance of the pig compared to rodents [11, 12]. Furthermore, pigs lack functional BAT with uncoupling protein 1, which eliminates this variable when measuring EE [13]. The Göttingen Minipig is a genetically and microbiologically well-defined breed that is commonly used in pharmacology and toxicology studies [14]. Furthermore, it is globally and commercially available (https://minipigs.dk/). Indirect calorimetry is the gold-standard method for assessing EE in the clinic [15]. Many studies have investigated the effect of nutritional interventions on EE in pigs, yet few have assessed pharmacological interventions [16–18], and no thorough validation studies have been conducted using positive controls.

If no direct measurements of EE are available, EE can be estimated through predictive equations based on demographic and anthropometric parameters like the Harris-Benedict equation [19] and on body composition parameters like the Cunningham equation [20].

The primary aim of the current study was to validate the Göttingen Minipig as a model for assessing the effects of phenotypic and pharmacological interventions on EE and substrate utilization based on respiratory exchange ratio (RER). The secondary aim was to establish predictive equations in Göttingen Minipigs for resting metabolic rate (RMR) based on demographic, anthropometric, and body composition parameters.

## Methods

All experiments were approved by the Animal Experiments Inspectorate, Ministry of Environment and Food, Copenhagen, Denmark (2018-15-0201-01414:C03 and 2022-15-0201-01350:C01). The in vivo part of the present study took place at the animal facilities of Novo Nordisk A/S (Ganloese, Denmark).

### Study design, animals, and housing

**Fig. 1:**
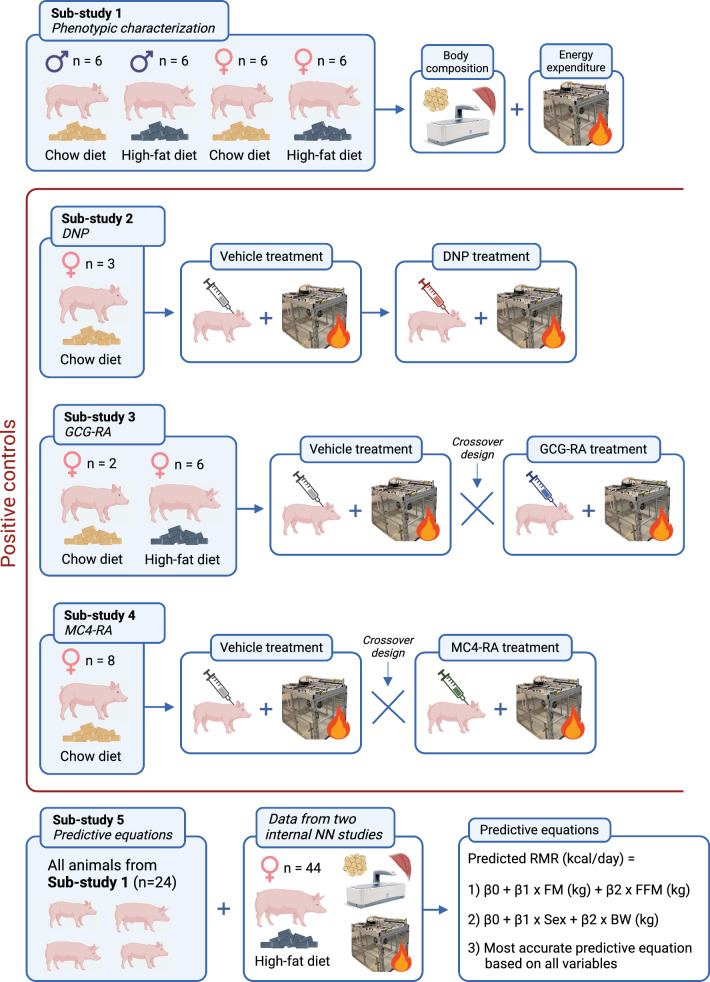
Study designs Study design for all five sub-studies. *DNP* 2,4-dinitrophenol, *GCG-RA* glucagon receptor agonist, *MC4-RA* melanocortin 4 receptor agonist, *NN* Novo Nordisk A/S, *RMR* resting metabolic rate, *FM* fat mass, *FFM* fat-free mass, *BW* body weight

The present study compromises five sub-studies (Fig. 1) using Göttingen Minipigs from the AAALAC accredited facility at Ellegaard Göttingen Minipigs A/S, Dalmose, Denmark in all studies (https://minipigs.dk/). The animals were fed either chow (Altromin 9023, Brogaarden, Denmark) or a high-fat diet (HFD) (Foulum, Aarhus University, Denmark) (Supplementary material 2) and had ad libitum access to water. They were housed individually at 21° C on a 12-h light/dark cycle. The baseline characteristics and diet regimen for each study are summarized in Table 1.

**Table 1:** Baseline characteristics of the minipigs in all five sub-studies

Baseline characteristics			Sex	Diet	n	Age	BW
Sub-study 1: *Phenotypic characterization* (total n = 24)			Male	Chow	6	8.6 (0.2)	19.3 (1.3)
			Male	HFD	6	8.7 (0.2)	28.2 (4.2)
			Female	Chow	6	8.0 (0.1)	21.3 (1.4)
			Female	HFD	6	8.1 (0.1)	45.9 (4.7)
Sub-study 2: *DNP study* (total n = 3)			Female	Chow	3	17.3 (1.0)	35.1 (1.9)
Sub-study 3: *GCG-RA study* (total n = 8)			Female	Chow	2	11.4 (0.0)	20.4 (3.0)
			Female	HFD	6	10.5 (0.1)	55.8 (3.4)
Sub-study 4: *MC4-RA study* (total n = 8)			Female	Chow	8	9.3 (0.4)	19.8 (0.5)
Sub-study 5: *Predictive equations* (total n = 68)		All sub-study 1	Male/female	Chow/HFD	24	8.3 (0.4)	28.7 (11.1)
		NN internal study A	Female	HFD	21	20.4 (1.2)	77.4 (11.2)
		NN internal study B	Female	HFD	23	17.8 (0.8)	69.6 (6.0)

Baseline characteristics of all five sub-studies. Values of age (months) and body weight (kg) (BW) are reported as mean (SD). HFD: high-fat diet

Sub-study 1 (n = 24) characterized EE in both chow- and HFD-fed males and females. Sub-study 2 (n = 3) assessed the impact of DNP on EE. During this study, the pigs were placed in metabolic chambers for 2 consecutive days, and all pigs received a dose of the vehicle on the first day and DNP on the second day. Sub-study 3 (n = 8) examined the impact of a glucagon receptor analog (GCG-RA) on EE. The pigs were kept in the metabolic chambers for 2 days, and this study was conducted as a cross-over study, where one-half of the pigs were dosed with vehicle and the other half with GCG-RA on the first day, and the other way around on the second day. Sub-study 4 (n = 8) evaluated the effect of an MC4-RA on EE. The pigs were kept in the metabolic chambers for 5 days since the drug was up-titrated over 4 days based on previous *in-house* experience. This study also had a cross-over design like that of sub-study 3. Lastly, in sub-study 5 (n = 68), we combined the data obtained in sub-study 1 with baseline data from two studies conducted internally at Novo Nordisk A/S (not further described here) to establish a predictive equation for RMR based on various parameters such as sex, fat mass (FM), and fat-free mass (FFM). A summary of the design of all five sub-studies is illustrated in Fig. 1.

### Intravenous access and setup for infusion and/or blood sampling

**Fig. 2:**
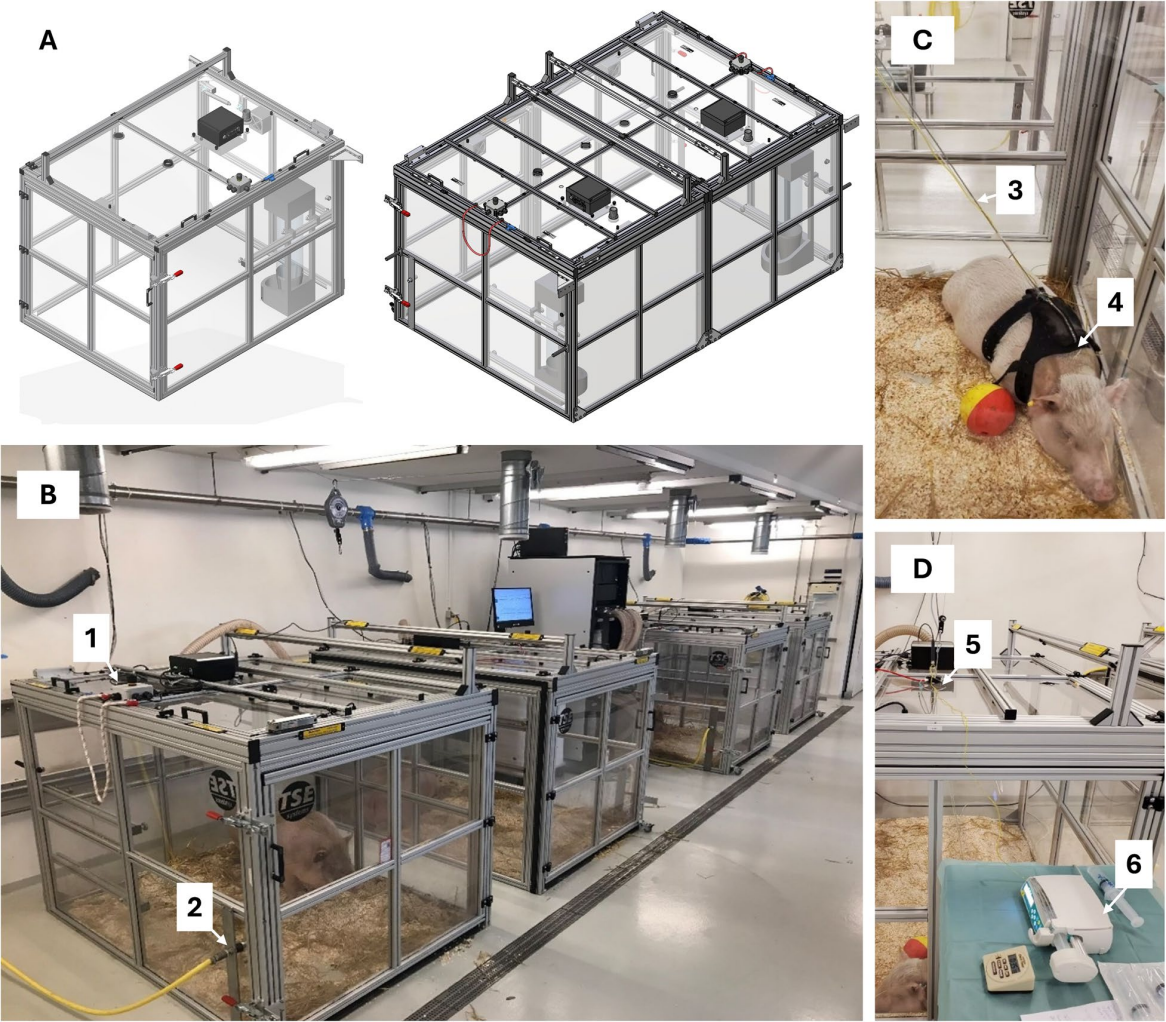
Schematic drawing and images of the metabolic chambers Indirect calorimetry chambers for pigs. The metabolic chambers are an experimental setup and not housing conditions. 2**A**. Schematic representation of the indirect calorimetry chamber, illustrating single/individual pig housing and the assembly of two chambers for larger pigs/groups. 2**B**. Photograph depicting four chambers with pigs inside, highlighting: Arrow A in 2**B** The integrated safety box for the lid. Arrow B in 2**B** Freshwater supply. 2**C** and **D**. Images demonstrating the study setup for infusion or blood sampling during EE measurements without disturbing the pigs or opening the chamber door: Arrow C in 2**C** Infusion tubes passing through the air intake and connected to the central venous catheter located in the backpack. Arrow D in 2**C** Backpack housing and safeguarding of the catheter. Arrow E in 2**D** The air intake opening. Arrow F in 2**D** Infusion pump enabling the continuous infusion of compounds

In some of the studies, central-venous access was needed for intravenous (IV) infusion of the test compounds. This was obtained by implanting a central-venous catheter in either the jugular vein through the ear vein or in the caudal caval vein as previously described [21] and the anesthesia protocol is provided in Supplementary material 2. The catheterization of the pigs allowed for continuous infusion and/or blood sampling during the measurements of EE in the metabolic chambers. An example of the setup is provided in Fig. 2C–D.

### Assessment of body composition

Body composition was assessed by dual-energy X-ray absorptiometry (DXA) scan in sub-study 1 and in the two internal NN studies used in sub-study 5 using a GE Lunar Prodigy Pro DXA scanner (Scanex Medical Systems A/S, Hørsholm, Denmark). The equipment was calibrated daily in accordance with manufacturing specifications, and the DXA estimates of total body weight (BW) were compared to the measured BW by a whole-body floor scale on the same day to ensure accuracy. The procedure was conducted while the pigs were anesthetized with 50 mg/kg tiletamine-zolazepam mixture (Zoletil 50 Vet, Virbac, Silkeborg, Denmark) with added xylazine, ketamine, and butorphanol (a special mixture for minipigs) in accordance with Christoffersen et al. [21], and placed in ventral recumbency with the hind legs and front legs stretched backwards. The body composition estimates include total BW, FM, FFM, and bone mass. FM% and FFM% were then calculated.

### Measurement of energy expenditure using indirect calorimetry

All pigs were subjected to a minimum of two time-separated 2-h sessions in the chambers to acclimatize them to the metabolic chambers and limit the impact of environmental changes on EE. No activity measurements were used in these studies.

#### Energy expenditure chambers

The hardware used was custom-built chambers from TSE Systems (Berlin, Germany) (Custom-build—6076 RespChamber). Each chamber has the following inner dimensions (L 1.64 m; W 1.21 m; H 1.24 m), resulting in a volume of 2.47 m^2^. Depending on the size of the subject and the length of the study, one chamber can be used alone, or two chambers can be assembled into one larger chamber to accommodate larger pigs or several smaller pigs (Fig. 2A). The chambers are made of plexiglass and have a drinking nipple for freshwater intake. Food can be administrated through the fresh-air hole at the top of the box and straight onto the floor. The door can also be opened and food can be placed in the feed trough, but the system then requires 15 min to return to equilibrium. The opening for fresh air intake is located at the top of the chamber, and two fans enable the distribution of fresh air throughout the chamber. The lid has a built-in safety box that measures nine different parameters such as carbon dioxide (CO_2_), hydrogen sulfide (H_2_S), ammonia (NH_3_), temperature, humidity, and lid status (open or closed). If one of these measurements reaches the lower or upper predefined threshold, the lid automatically opens to allow fresh air to enter the box.

The metabolic measurement system connected to the TSE chambers is an Omnical system from Maastricht Instruments (Maastricht, The Netherlands) that is used in human studies and was rebuilt for the pigs. This system measures the O_2_ and CO_2_ content of the ingoing and outgoing air from the chambers. The system shows the real-time data of oxygen uptake (VO_2_), carbon dioxide production (VCO_2_), and RER (VCO_2_/VO_2_) in addition to environmental parameters like temperature and humidity in both the cage and the incoming air. After each measurement, the exact real-time values of experiment time, EE (calculated by the Weir equation), RER, VO_2_, and VCO_2_ were saved in a data file, along with ambient pressure, relative humidity, temperature, fractional inspired oxygen, fractional content oxygen, flow volume, fractional inspired CO_2_ and fractional content CO_2_. The system accuracy is 1.0% ± 2.0% SD or better for VO_2_ and VCO_2_. The flow rate was set to 270 L/min in all experiments. Before each experiment, an automatic calibration was performed using calibration gas with a 1.0% accuracy (18.0% O_2_ and 0.8% CO_2_). During the experiment, either calibration gas or nitrogen was measured every 16 min for calibration purposes. We also performed a methanol burn test before and after each experiment to ensure that the system measurements fell within the theoretical values of the methanol.

#### Methodological considerations

The setup using indirect calorimetry estimates total EE, which compromises RMR, diet-induced thermogenesis (DIT), and activity-related EE (AEE) [22]. Each of these parameters can be the primary endpoint, depending on the scope of the study. In many cases, the effect on RMR is in scope, and therefore, the aim is to limit or monitor the impact of DIT and AEE. The variation in DIT between the animals can be limited by fasting the animals or feeding them a similar meal, and monitoring AEE using video analysis [23] or accelerometers [24] enables AEE to be quantified and accounted for when the data are analyzed. Changes in the environment or other factors that can impact the animals’ activity or stress levels can significantly influence EE. Therefore, it is essential to have an acclimation period for the animals in the metabolic chambers to familiarize them with the environment and minimize potential stress. It is also important to restrict human access to the animal room during measurements to avoid disturbances.

Similar to the RMR, the effect of pharmacological drugs is, in our experience, most accurately assessed during the resting period, where the measurements are minimally confounded by activity, varying states of arousal/stress, and food intake/digestion. However, the data analysis depends on the specific research question and the pharmacokinetic properties of the tested compounds. In sub-study 1, we analyzed EE during the night period as we were interested in comparing RMR between the groups. In sub-study 3, we investigated the acute effect of a relatively short-acting EE-elevating drug and thus the data were analyzed for the period immediately after the dosing, where the drug exposure was considered relevant, which again underscores the importance of selecting the appropriate timeframe for analysis. When analyzing EE, and if feasible, we recommend accounting for body composition, e.g., FM and FFM. This is done by adding the body composition parameters to the data analysis as covariates [25], although more simple anthropometric measures can also be used.

### Description of the sub-studies

#### Sub-study 1 (phenotypic characterization)

The pigs (n = 24) were divided into four groups: males (n = 6) and females (n = 6) fed a restricted amount of chow, as well as males (n = 6) and females (n = 6) fed an increasing amount of HFD until ad libitum feeding was established two weeks before the end of the trial. At the start of the feeding trial, the animals were approximately 5–6 months old, and the intervention lasted 3 months. After the feeding period, the animals underwent a 22-h measurement of EE in the metabolic chambers. During the EE measurements, all animals were given a standardized 320 g of chow to minimize variations in DIT between chow and HFD. After the EE measurements, body composition was assessed using a DXA scan the following day.

The RMR and RER were calculated from 12 PM to 5 AM since the pigs were expected to rest during this period.

#### Sub-study 2 (DNP)

This study was conducted as an initial pilot study, but since the data were already conclusive with n = 3 due to the large effect size, we decided not to include more animals in this study. The pigs (n = 3) were placed in the chambers on the morning of the first experimental day and given their daily meal. After at least 6 h acclimation in the chambers and 2 h before the onset of the dark phase, the pigs were hooked up with extension tubes, allowing IV infusion of vehicle/DNP from outside the chamber. The pigs were dosed with vehicle (sterile 0.9% saline) on Day 1 and DNP (Sigma Aldrich, St. Louis, MO, USA, 12 mg/kg in total, formulated in a concentration of 10 mg/ml in sterile 0.9% saline) on Day 2, and both treatments were given as a 30-min IV infusion through the implanted central-venous catheter (1.2 ml/kg/30 min). This 30-min IV dose regimen was selected based on previous *in-house* experience to achieve a relevant exposure level while avoiding an exposure peak that could give rise to the typical and often severe DNP-related side effects [18]. After the infusion, the catheter was flushed with sterile saline and closed. The pigs were then left undisturbed overnight with measurements of EE and RER. We excluded the first 1 h after the infusion from the analysis. The EE and RER were calculated from 6 PM to 6 AM the following morning since the pigs were expected to have received sufficient exposure to the compound and adequate rest during this period.

#### Sub-study 3 (GCG-RA)

Eight pigs were included in this study based on an expected effect size of approximately 5% and limited knowledge on variability of response. The pigs (n = 8) were placed in the chambers before noon on the day before the experiment to allow them to acclimate to the chambers and were given their daily meal at around 1 PM. The study had a cross-over design, where half of the pigs were dosed with the vehicle, and the other half with GCG-RA on the first day of the study with cross-over to the opposite treatment on the second day of the study. The test item used in the present study was a GCG-RA synthesized at Novo Nordisk A/S with a slightly improved in vitro potency on the human glucagon receptor compared to native glucagon and a slightly longer half-life (1.2 h after IV bolus injection) (data not shown). The dosing was administrated as an IV bolus injection (0.8 nmol/kg, 0.05 ml/kg) followed by a 45-min IV infusion in a dose of 1.6 nmol/kg/min (0.018 ml/kg/min). This dosing regimen was selected based on literature data of the effects of native glucagon on EE in humans [5] and pigs [26], together with internal Novo Nordisk A/S pharmacodynamic studies on the GCG-RA to quickly achieve an adequate exposure level. To avoid disturbing the pigs and opening the chambers during the dosing period, we hooked the pigs up to extension tubes, which allowed the IV infusion of vehicle/GCG-RA from outside the chamber. After the chamber was closed, the pigs were given an infusion with sterile 0.9% saline (0.01 ml/kg/min) for approx. 1.5 h to allow them to settle and the chamber to equilibrate again before the dosing with vehicle/GCG-RA. Following the 45-min infusion period, the pigs were allowed a washout period by infusion with sterile 0.9% saline (0.01 ml/kg/min) for approx. 1 h so that the effects on EE and RER could be followed after the infusion stopped. On each study day, the pigs were fed their daily meal after the procedure, at around 1 PM, to avoid any confounding effects of feeding on the EE and RER data. The EE and RER were calculated in the period from 20 to 45 min of the infusion period since this allowed a short period for the effect to kick in and corresponded to the period with steady-state exposure levels.

#### Sub-study 4 (MC4-RA)

Eight pigs were included in this study based on an expected effect size of approximately 5% and limited knowledge concerning variability in the response. The pigs (n = 8) were placed in the chambers before noon on the day before the experiment to allow them to acclimate to the chambers. The study was conducted with a cross-over design, with each pig being studied in 2 rounds with 1 week of washout/recovery between rounds. In each round, half the pigs were dosed with vehicle and the other half were dosed with MC4-RA (LY2112688, described in Greenfield et al. [27] and prepared at Novo Nordisk A/S) for 4 consecutive days with cross-over to the opposite treatment in the second study round. The compound was formulated in 50 mM phosphate, 70 mM sodium chloride, 0.007% polysorbate 20, pH = 5, and this formulation also served as vehicle. The pigs were dosed subcutaneously twice daily at approx. 8.30 AM and 5.30 PM with increasing doses of the MC4-RA (0.03, 0.06, 0.1, and 0.1 mg/kg on the 4 days, respectively, corresponding to a dose of volume 0.003–0.01 ml/kg). This dose regimen was chosen to avoid side effects based on previous experience with this compound in pigs. On the morning of each study day, the pigs were fed their daily meal and were exercised outside the chamber for 30 min while the chamber was cleaned, and afterward, the chambers were allowed 1 h to equilibrate again before the measurements were considered valid. Thus, the period from approx. 8 AM–10 AM was excluded from the analysis. The EE and RER were calculated from midnight to 5 AM the following morning on all study days, but only the data from the last night, when the 0.1 mg/kg dose had been given four times, were used since the pigs here were expected to be sufficiently exposed to the compound and resting.

#### Sub-study 5 (predictive equation)

All the data from the minipigs (n = 24) in sub-study 1 were included in sub-study 5. Also included were baseline data obtained from two studies involving female Göttingen Minipigs (n = 21 and n = 23) fed HFD, which were conducted internally at Novo Nordisk A/S. In these two studies, the animals were subjected to similar conditions in the metabolic chambers and subsequently the DXA scan. For the predictive equations, we identified the lowest 30-min interval during the night (12 PM–5 AM) for each animal as RMR since this interval would be the closest approximation to eliminating activity. RMR, RER, body composition, demographic, and anthropometric variables were utilized to establish the most accurate predictive equations.

### Euthanasia

After the studies, the pigs were either transferred to the colony and used in other studies or euthanized by deep anesthesia with Zoletil mixture (Supplementary material 2) followed by exsanguination.

### Statistical analyses

For sub-studies 1–4, the relevant period was summarized and visualized using XY graphs for EE and RER. The mean value of the relevant period was calculated to test for significant differences between groups/treatments in EE or RER, and a paired t-test or a two-way ANOVA with Šídák’s multiple comparisons test was employed. In addition, in sub-study 1, Adjusted EE (Adj_EE) was computed using FM and FFM as covariates, and subsequently, a two-way ANOVA with Šídák’s multiple comparisons test was employed.

In sub-study 5, predictive equations were established using multiple linear regression with the dependent variable RMR (lowest 30-min EE interval during the night period) and regressors based on three models: (1) FM and FFM, (2) sex and BW, and (3) the most accurate predictive equation based on all included variables. The accuracy of the models was assessed using the adjusted R-squared (Adj_R^2^) and residual standard error (RSE). A *P*-value < 0.05 was considered significant.

The data were analyzed and visualized using GraphPad Prism version 10.1.2 for Windows, developed by GraphPad Software, Boston, Massachusetts, USA (www.graphpad.com).

## Results

In sub-study 1 and subsequently sub-study 5, one pig was excluded from the data analysis because of technical problems with the DXA scan. In sub-study 4, two pigs were excluded from the data analysis because they received incorrect dosing on the third dosing day, which affected the results on the final and fourth study day, where the dose had been titrated to a relevant level.

### Sex, diet, and body composition influenced EE and/or RER

**Fig. 3:**
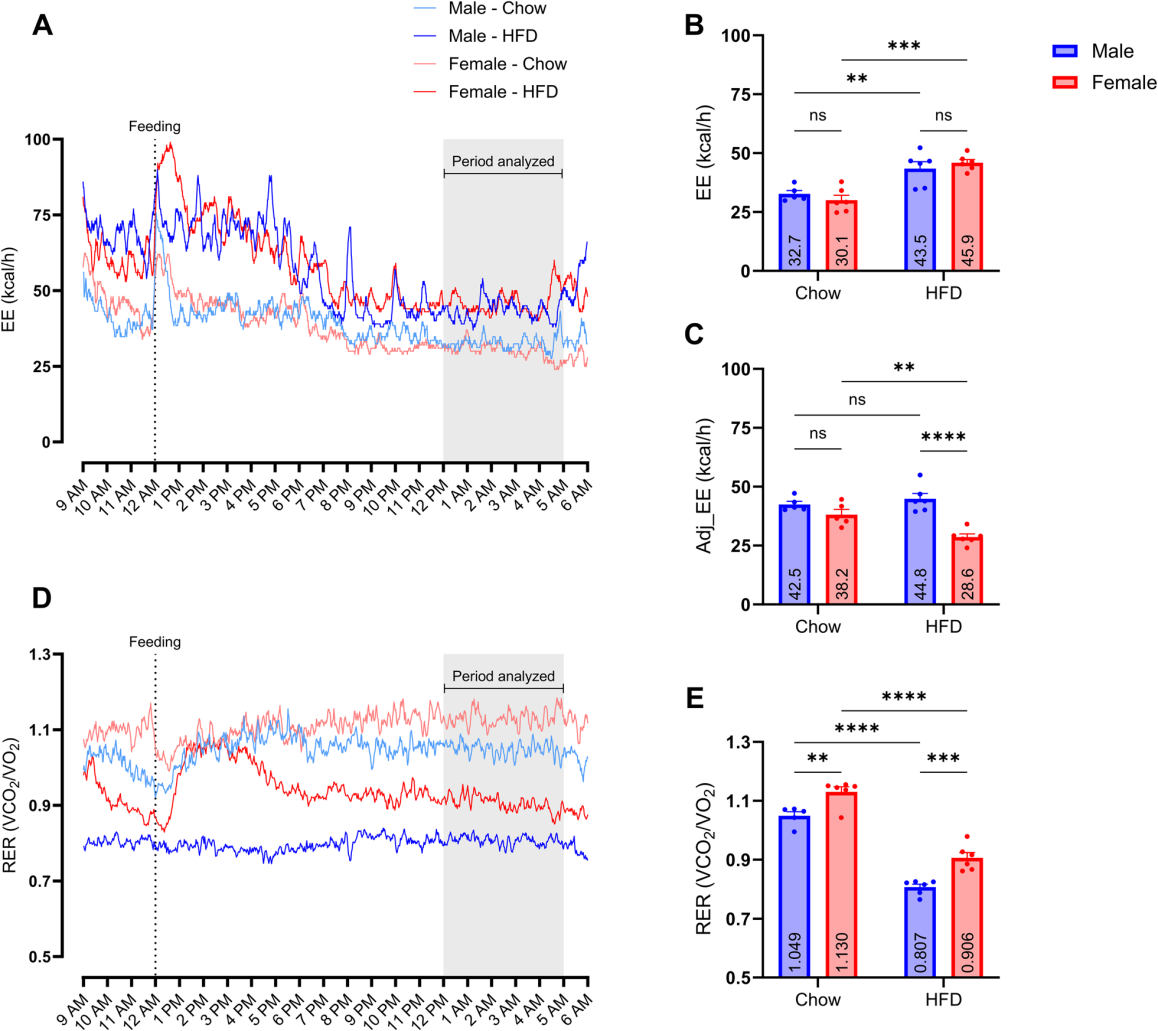
Effect of sex, diet, and body composition on EE and RER Sub-study 1: Phenotypic characterization of EE and RER by comparing diets (chow/HFD) and sex (male/female). A + D: Mean value EE and RER visualized during the relevant measurement period. B + C + E: Calculated mean ± SEM and individual values for EE, EE adjusted for FM and FFM (Adj_EE), and RER per group. The groups were compared using a two-way ANOVA with Šídák’s multiple comparisons test. Significant comparisons between groups are reported as **p < 0.01; ***p < 0.001; ****p < 0.0001; *ns* not significant, *EE* energy expenditure, *RER* respiratory exchange ratio, *HFD* high-fat diet

Energy expenditure was significantly influenced by diet (chow/HFD, *P*-value < 0.0001), which was primarily due to differences in body sizes, and no significant effect of sex was found. Males fed HFD had a significantly higher EE, compared to males fed chow, by 10.8 kcal/h [95% CI: 2.4–19.1] (*P*-value: 0.0085) and females fed HFD had a significantly higher EE, compared to females fed chow, by 15.9 kcal/h [95% CI 7.9–23.8] (*P*-value: 0.0001) (Fig. 3B). Adj_EE was significantly influenced by sex (male/female, *P*-value < 0.0001) and not diet. Compared to females fed HFD, female pigs fed chow had a significantly higher Adj_EE by 9.6 kcal/h [95% CI 2.2–16.9] (*P*-value: 0.0079). Female pigs fed HFD, compared to males fed HFD, had a significantly lower Adj_EE by 16.2 kcal/h [95% CI 9.3–23.2] (*P*-value < 0.0001) (Fig. 3C). Respiratory exchange ratio was significantly influenced by both diet (chow/HFD) and sex (male/female). Males fed HFD had a significantly lower RER, compared to males fed chow, by 0.242 [95% CI 0.180–0.304] (*P*-value < 0.0001). Females fed HFD also had a significantly lower RER, compared to females fed chow, by 0.224 [95% CI 0.165–0.283] (*P*-value < 0.0001). Males fed HFD had a significantly lower RER, compared to females fed HFD, by 0.099 [95% CI 0.040–0.158] (*P*-value: 0.0007). Males fed chow also had a significantly lower RER, compared to females fed chow, by 0.081 [95% CI 0.019–0.143] (*P*-value: 0.0075) (Fig. 3E).

### DNP treatment increased EE and decreased RER significantly

**Fig. 4:**
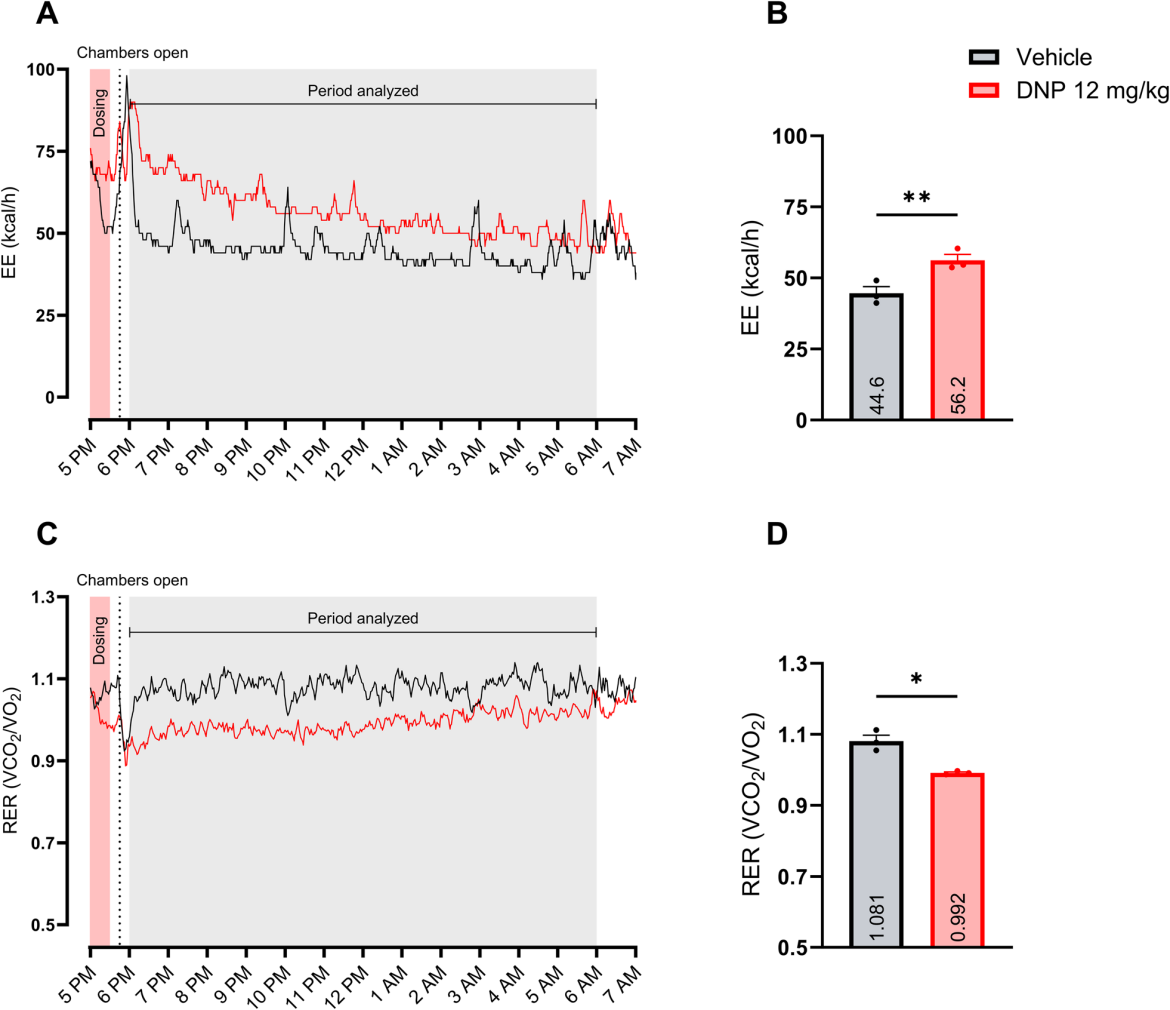
DNP treatment effect on EE and RER Sub-study 2: Evaluation of DNP (12 mg/kg) on EE and RER compared to vehicle. A + C: Mean value EE and RER visualized during the relevant measurement period. “Chambers open” refers to the opening of the metabolic chambers to remove the infusion setup after dosing. B + D: Calculated mean ± SEM and individual values for EE and RER per treatment in the period analyzed. The treatment effect was analyzed using a paired t-test. Significant treatment comparisons are reported as *p < 0.05; **p < 0.01. *EE* energy expenditure, *RER* respiratory exchange ratio

Treatment with DNP compared to vehicle significantly increased EE by 11.6 kcal/h [95% CI 9.8–13.5] (*P*-value: 0.0014), corresponding to a 26.1% increase (Fig. 4B). Treatment with DNP, compared to vehicle, significantly decreased RER by 0.090 [95% CI 0.011–0.168] (*P*-value: 0.0391), corresponding to an 8.3% decrease (Fig. 4D).

### GCG-RA treatment increased EE and RER significantly

**Fig. 5:**
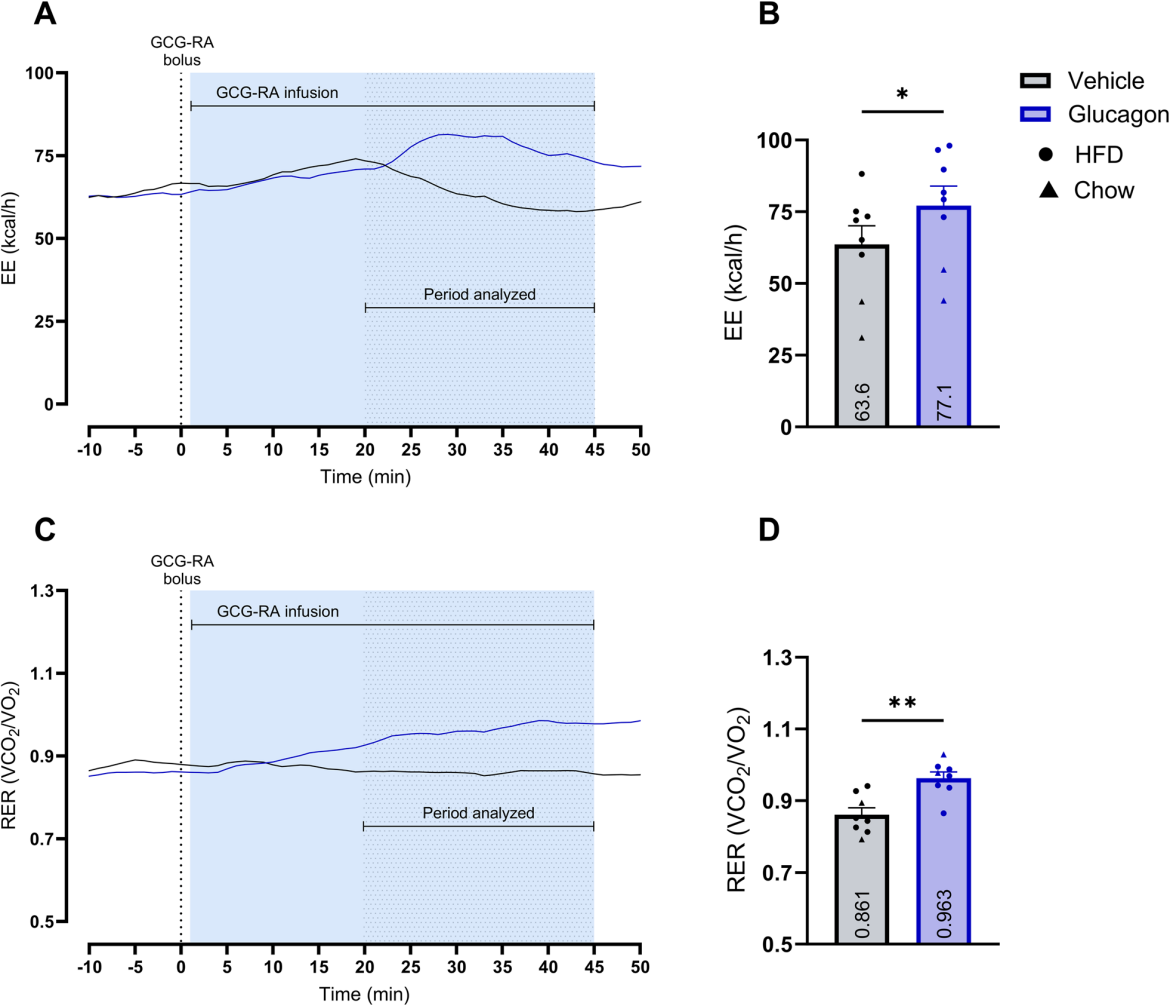
GCG-RA treatment effect on EE and RER Sub-study 3: Evaluation of a GCG-RA on EE and RER compared to vehicle. A + C: Mean value EE and RER visualized during the relevant measurement period. B + D: Calculated mean ± SEM and individual values for EE and RER per treatment in the period analyzed. The treatment effect is analyzed using a paired t-test. Significant treatment comparisons are reported as *p < 0.05; **p < 0.01. *EE* energy expenditure, *RER* respiratory exchange ratio, *HFD* high-fat diet

Treatment with GCG-RA compared to vehicle significantly increased EE by 13.6 kcal/h [95% CI 0.1–27.0] (*P*-value: 0.0491), corresponding to a 21.3% increase (Fig. 5B). GCG-RA, compared to vehicle, also significantly increased RER by 0.102 [95% CI 0.040–0.164] (*P*-value: 0.0061), corresponding to an 11.8% increase (Fig. 5D).

### MC4-RA treatment increased EE significantly and tended to decrease RER

**Fig. 6:**
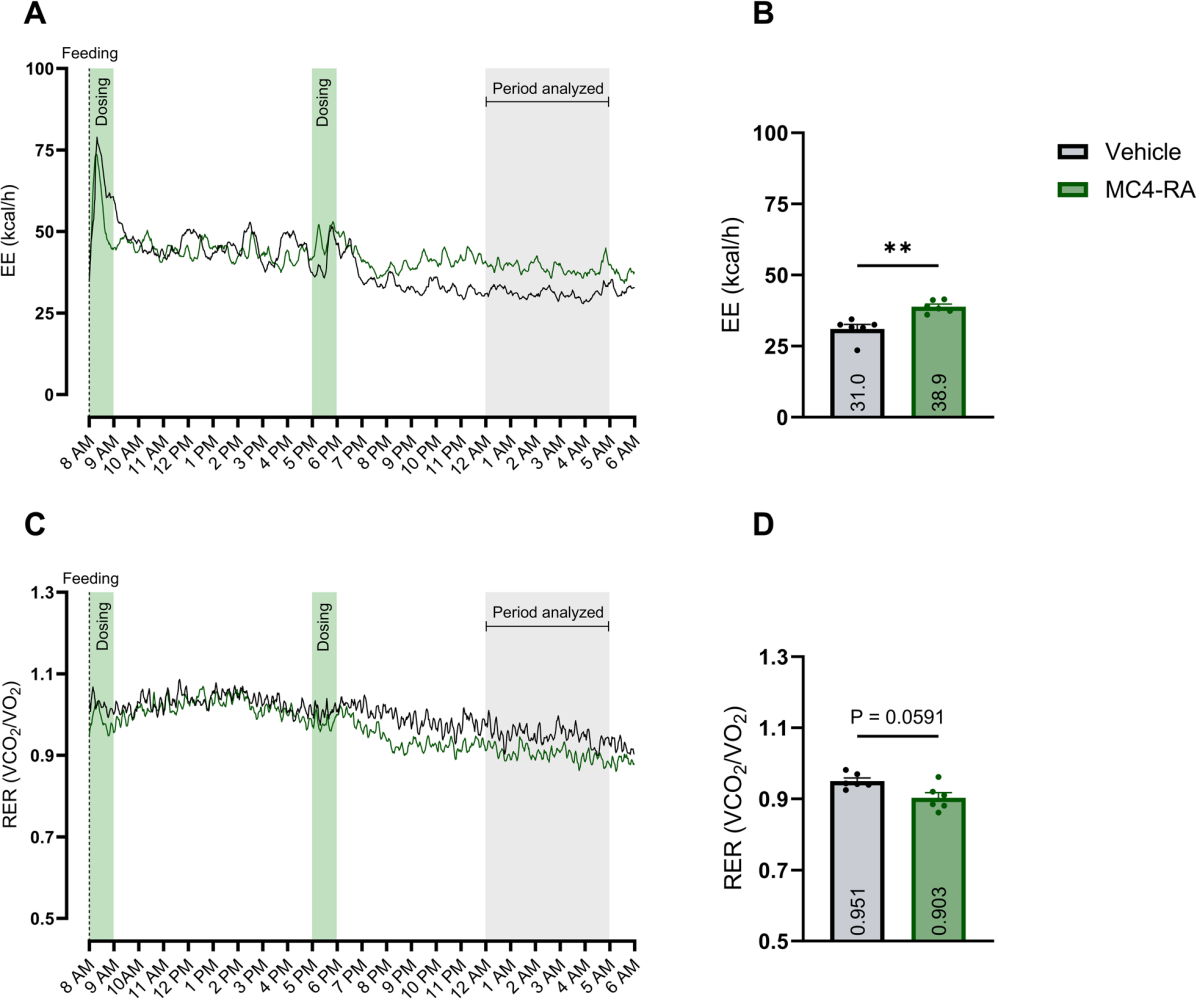
MC4-RA treatment effect on EE and RER Sub-study 4: Evaluation of an MC4-RA (0.1 mg/kg) on EE and RER compared to vehicle. A + C: Mean value EE and RER visualized during the last day of dosing. B + D: Calculated mean ± SEM and individual values for EE and RER per treatment in the period analyzed. The treatment effect is analyzed using a paired t-test. Significant comparisons between treatments are reported as *p < 0.05; *P* numeric P-value. *EE* energy expenditure, *RER* respiratory exchange ratio

Treatment with MC4-RA significantly increased EE compared to vehicle on the last night after the last dose by 7.9 kcal/h [95% CI 4.8–11.0] (*P*-value: 0.0013), corresponding to a 25.4% increase (Fig. 6B). MC4-RA, compared to vehicle, did not significantly affect RER, but a tendency for a decrease was observed (0.047 [95% CI − 0.003–0.097] (*P*-value: 0.0591)) (Fig. 6D). Average 5 h nighttime data from all days can be found in Supplementary material 1.

### Three predictive equations were established and resulted in acceptable accuracy

**Fig. 7:**
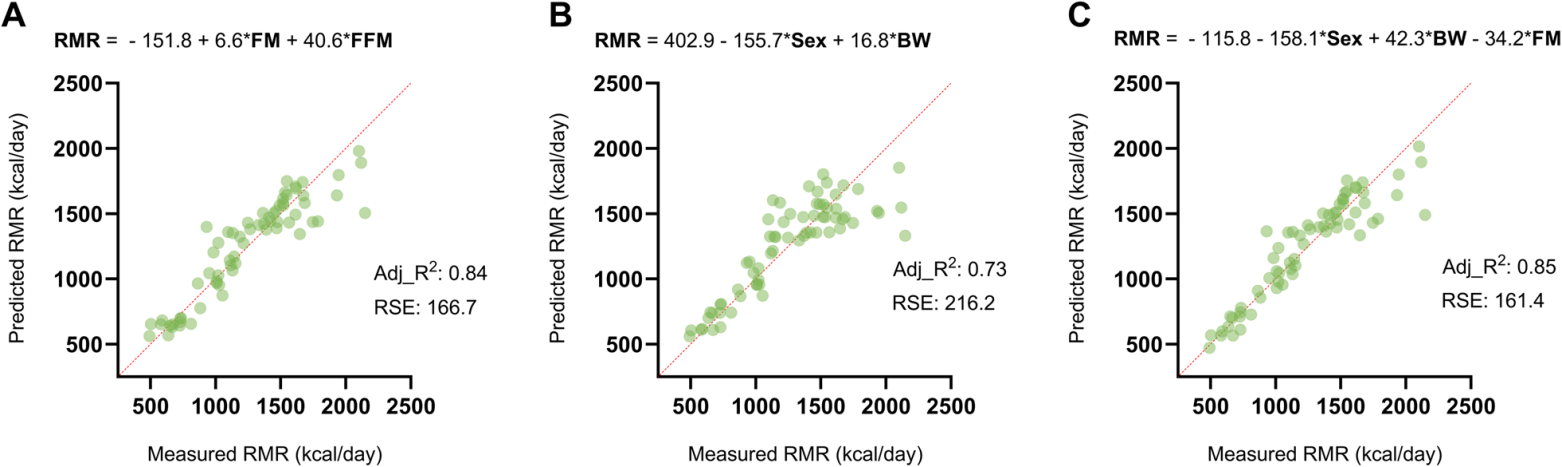
Accuracy of predictive equations Sub-study 5: Predictive equations for RMR based on (A) body composition (FM and FFM), (B) anthropometric measurements (sex and BW), and (C) most accurate predictive equation based on both body composition and anthropometric measurements. Accuracy is reported by the adjusted R^2^ (Adj_R^2^) and the mean residual standard error (RSE). *RMR* resting metabolic rate, *FM* fat mass, *FFM* fat-free mass, *BW* body weight

The body composition predictive equation based on FM and FFM resulted in Adj_R^2^ of 0.84 and a RSE of 166.7 kcal/day (Fig. 7A). The anthropometric predictive equation based on sex and BW resulted in Adj_R^2^ of 0.73 and an RSE of 216.2 kcal/day (Fig. 7B). Lastly, the most accurate predictive equation based on all variables used sex, BW, and FM and resulted in Adj_R^2^ of 0.85 and an RSE of 161.4 kcal/day (Fig. 7C).

## Discussion

The present study examined a new setup for indirect calorimetry measurements in Göttingen Minipigs. The system was validated by measuring differences in EE and RER based on phenotypes and expected changes in EE and RER after the administration of drugs with a known effect on these parameters in humans. Three predictive equations for RMR were established, showcasing acceptable accuracy compared to the equations used in the clinic, and with the potential for further development when more data are obtained.

Although it is difficult to make comparisons between studies, the EE data obtained in the current study are within a similar range as observed in sexually mature Göttingen Minipigs [28] but higher than observed in Yucatan minipigs [29].

In sub-study 1, EE was significantly influenced by diet, which was expected due to differences in body sizes, and this effect of body size was also observed in a study in Göttingen Minipigs by Bollen et al. [28]. Adj_EE resulted in a significant influence of sex (Fig. 3B–C). Compared to males fed HFD, females that were fed HFD had a higher BW with more FM and less FFM and a lower Adj_EE. In humans, while most of the variation in RMR can be explained by FFM, women seem to have a slightly lower RMR even when adjusted for body composition [30], which is consistent with our findings in minipigs. One might also speculate that the higher BW in the HFD-fed females could contribute to a reduced level of activity, which in turn might decrease EE. However, this would need to be verified by tracking activity levels in the metabolic chambers while assessing EE. As expected, RER was lower in the HFD-fed animals, indicating more fatty acid metabolism, as also shown by others [31, 32] (Fig. 3E). Females had a higher RER than males, suggesting less fatty acid oxidation or increased degree of lipogenesis in females, as also supported by their higher levels of FM compared to males. Bollen et al. [28] also observed a similar sex difference in RER after sexual maturation.

In sub-study 2, DNP demonstrated a significant increase in EE by 26.1% despite sample size of only three pigs (Fig. 4B). This increase is of similar magnitude to what the current authors earlier described in minipigs [18], where a 33.2% increase in EE was observed with 10 mg/kg measured by the isotope ^13^C-bicarbonate method. In the study by Cutting et al. [4], DNP increased EE by 20%-40% in humans dose-dependently. Regarding RER, the present study revealed a significant decrease in the DNP-treated animals (Fig. 4D), which is consistent with the findings of both aforementioned studies. In mice, the impact of DNP on total EE was influenced by the housing temperature, and the effect was only observed at thermoneutrality (30 °C), presumably due to a counterregulatory reduction in BAT activation at 22 °C [33]. Additionally, no effect of DNP on RER was observed in the rodent study at either temperature, highlighting the translational challenges from rodents to humans.

In sub-study 3, GCG-RA demonstrated a significant increase in EE by 13.6 kcal/h despite a more pronounced interindividual variation in effect size as indicated by the confidence interval of 0.1–27.0 kcal/h (Fig. 5B). Additionally, one animal even displayed a decrease in EE by 15.2 kcal/h. It is important to note that when investigating a short-term EE change (minutes), the data are more sensitive to being influenced by activity, which was not measured. Ingram et al. [26] also confirmed the EE-increasing effect of glucagon in pigs, showing a mean increase of 26%, comparable to the 21.3% increase observed in the present study. In humans, glucagon administration can increase EE by 100–200 kcal/day, corresponding to approx. 5–10%, and seems to be affected by the prandial state, i.e., improved effect size seen in fasting individuals [7]. In rats, native glucagon has also been shown to increase EE up to 50% dose-dependently, which is greater than what is observed in pigs and humans [34]. In the present study, RER increased significantly with GCG-RA administration (Fig. 5D), confirming the results observed in humans [5]. This effect can be explained by the physiological effects of glucagon in releasing glucose into the bloodstream, thereby allowing for more carbohydrate utilization.

In sub-study 4, MC4-RA treatment led to an increase in EE, but only during the night period (Fig. 6A), highlighting the importance of studying pharmacological effects during both nighttime and daytime, since the effect during the day-time can be masked by activity, feeding, and general arousal, unless the effect is highly pronounced. Since the dosing in our study was up-titrated and should have been maintained at a steady state during the last day, in theory, we should have seen an effect during the daytime as well, which was not the case. As reported in Supplementary material 1, we observed a significant effect of the MC4-RA treatment from the first night after MC4-RA dosing and onwards when analyzing all five nightly periods (Supplementary material 1 Fig. 1A). Another study in obese Gottingen Minipigs found no differences in VO_2_ between vehicle and MC4-RA treatment, but those data were not corrected for the significant weight loss in the MC4-RA group [17]. Similar to our findings, but in a lesser magnitude, a clinical study by Chen et al. found that an MC4-RA (RM-493) increased RMR by 6.4% and lowered RER significantly [8]. The same MC4-RA also increased EE by 14% in diet-induced obese non-human primates measured by double-labeled water [35]. RM-493 also promoted increases in EE of approx. 10% and decreases in RER in diet-induced obese mice [36].

In sub-study 5, three predictive equations were established with acceptable precision of Adj_R^2^ values from 0.73 to 0.85 (Fig. 7A–C), which is in line with similar predictive equations used in the clinic that generally have R^2^ values ranging from 0.36 to 0.84 [37]. In the present study, the bias or RSE ranged from 161 to 216 kcal/day, depending on which equation was used. When expressed as a percentage of RMR, this RSE ranged from 13 to 17%, indicating a relatively high level of error. For all three predictive equations, we also observed a tendency regarding the highest quartile for RMR to be underestimated compared to the measured RMR. The mean RSE measured in the clinic is around 160 kcal/day or under 10% of the average RMR [37]. These predictive equations can be further optimized when more data, including activity data, are obtained.

The current study is limited by the lack of activity monitoring, a small sample size of animals, and the practical restriction of having only four metabolic chambers, which limited the number of animals that could simultaneously participate in experiments. Future studies should aim at implementing activity monitoring in these types of studies in order to control for this variable when assessing the effect of new drug candidates. Additionally, assessing the effect of other positive control interventions, including some that reduce EE, could add to the validation provided in this paper.

## Conclusions

The current research presents new equipment and a method for measuring and analyzing EE in pigs (and other similar-sized animals) using indirect calorimetry. Sex, diet, and body composition influenced EE and/or RER comparable to what has been observed in humans. Additionally, the effect of three pharmacological interventions known to increase EE in humans and either decrease or increase RER (DNP, a GCG-RA, and an MC4-RA) showed similar effects in the Göttingen Minipigs, emphasizing the translational value of the model. Lastly, three predictive equations were established for RMR (body composition as well as demographic and anthropometric parameters, and with the inclusion of all variables), demonstrating acceptable accuracy compared to the ones used in the clinic. Overall, the Göttingen Minipigs proved to be a valuable animal model for testing drug candidates with effect on EE and RER. The use of Göttingen Minipigs could potentially improve the translational value of preclinical research related to EE and bridge the gap between rodent models and humans.

## Supplementary Information

Supplementary material 1.

Supplementary material 2.

## Data Availability

The datasets used and/or analyzed during the current study are available from the corresponding author on reasonable request.

## Abbreviations

Adj_EE: Adjusted energy expenditure
AEE: Activity-related energy expenditure
BAT: Brown adipose tissue
BW: Body weight
DIT: Diet-induced thermogenesis
DNP: 2,4-Dinitrophenol
DXA: Dual-energy x-ray absorptiometry
EE: Energy expenditure
FFM: Fat-free mass
FM: Fat mass
GCG-RA: Glucagon receptor agonist
HFD: High-fat diet
MC4-RA: Melanocortin 4 receptor agonist
RER: Respiratory exchange ratio
RMR: Resting metabolic rate
RSE: Residual standard error
VCO_2_: Carbon dioxide production
VO_2_: Oxygen uptake
