# Supplementary material for: Measuring energy expenditure in Göttingen Minipigs using indirect calorimetry: validation and methodological considerations
Source: Lab Anim Res. 2025 Feb 21;41:9. doi: 10.1186/s42826-024-00233-3 (PMC11843975; doi:10.1186/s42826-024-00233-3)
Supplement: Supplementary file 1 — Supplementary material 1. [file 42826_2024_233_MOESM1_ESM.docx]

**Additional file 1**

In sub-study 4, the effect of an MC4-RA on EE and RER was compared to a vehicle-treated group. The drug was up-titrated over four days, and the average 5 h night-time data from all days were calculated and analyzed (Additional file 1 Fig 1A-B).

Additional file 1 Fig. 1:


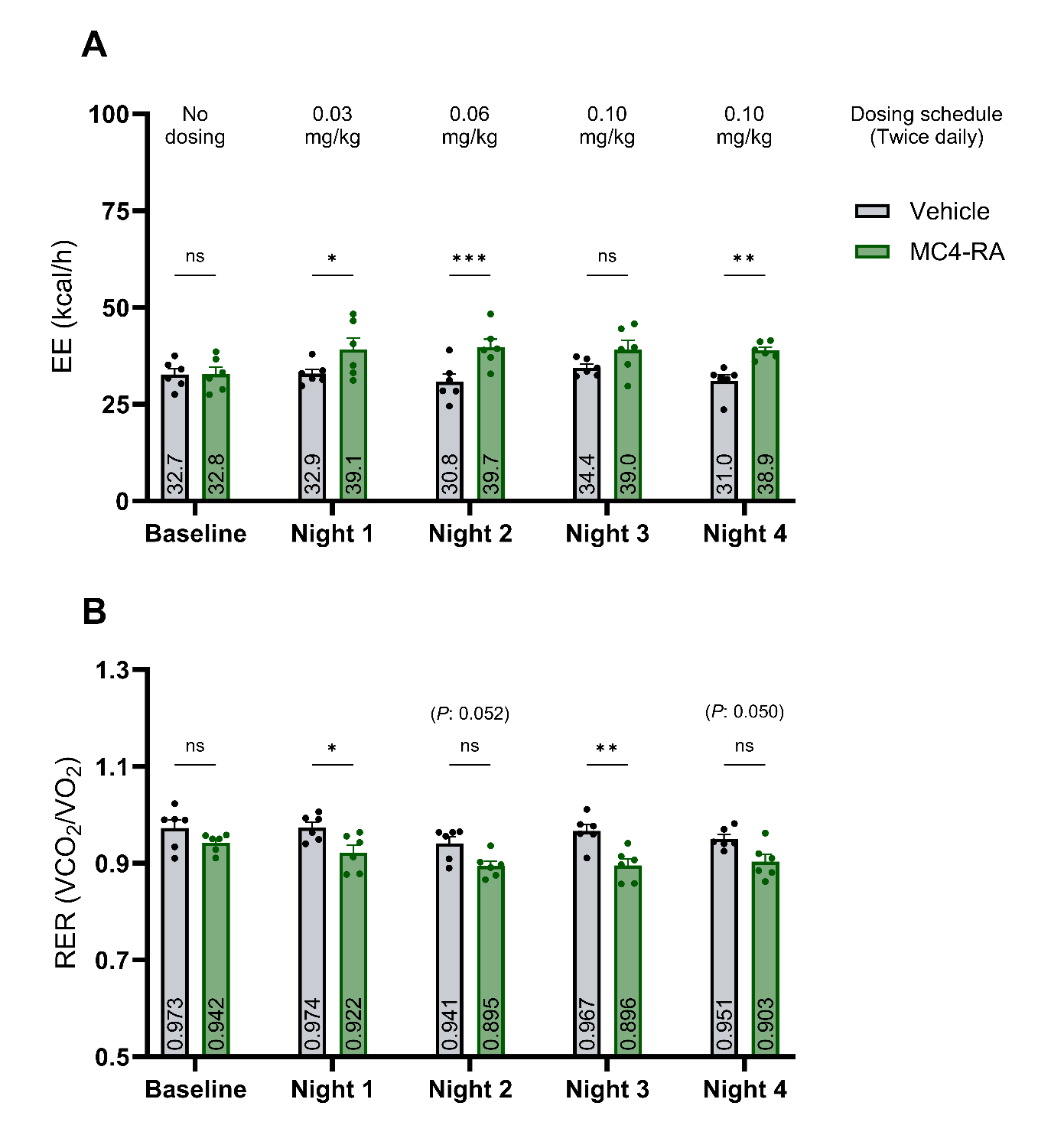


*Additional file 1 Figure 1: Sub-study 4: Evaluation of an MC4-RA (0.1 mg/kg) on EE and RER compared to vehicle. A-B: Calculated mean±SEM and individual values of EE and RER during the night period for all five days. The treatment effect is analyzed by a repeated measures two-way ANOVA with Šídák's multiple comparisons test. Significant comparisons between treatments are reported as *p<0.05; **p<0.01; ***p<0.001; ns: not significant; numeric p-values reported for P-values < 0.1. A: Dosing schedule reported. EE: energy expenditure; RER: respiratory exchange ratio.*
