# Supplementary material for: Measuring energy expenditure in Göttingen Minipigs using indirect calorimetry: validation and methodological considerations
Source: Lab Anim Res. 2025 Feb 21;41:9. doi: 10.1186/s42826-024-00233-3 (PMC11843975; doi:10.1186/s42826-024-00233-3)
Supplement: Supplementary file 2 — Supplementary material 2. [file 42826_2024_233_MOESM2_ESM.docx]

**Additional file 2**

Information about chow and high-fat diets

**Chow diet**: Altromin 9023, from Brogaarden, Denmark.

The formula is a cereal-based (soy, wheat, corn) fixed formula.

Total metabolizable energy content: 2.857 kcal/kg

Fat: 309 (11%) kcal/kg

Protein: 545 (19%) kcal/kg

Carbohydrates: 2.003 (70%) kcal/kg

**High-fat diet**: a specialized diet from Foulum, Aarhus University, Denmark

The formula is a mixture of wheat, barley, oat, fructose, turnip, chalk, monocalcium phosphate, soybean oil, lard, cholesterol, and minerals.

Total metabolizable energy content: 4.598 kcal/kg

Fat: 2213 (48%) kcal/kg

Protein: 491 (11%) kcal/kg

Carbohydrates: 1898 (41%) kcal/kg

Anesthesia for implementing central-venous catheters

Implantation of central-venous catheters was done in 18 h fasted animals under general anesthesia induced by 1 ml/10-15 kg of a mixture containing 125 mg zolazepam and 125 mg tiletamine (Zoletil® 50 Vet, ChemVet, Denmark), ketamine (1.25 mL Ketaminol® Vet 100mg/mL, MSD Animal Health, Denmark), xylazine (6.5 mL Rompun Vet, 20 mg/mL, Bayer A/S, Denmark) and butorphanol (2.5 mL Torbugesic®, 10 mg/mL, Scanvet, Denmark)). This was supplemented with 1/3 of the initial dose if needed.
